# *wblE2* transcription factor in *Streptomyces griseus* S4‐7 plays an important role in plant protection

**DOI:** 10.1002/mbo3.494

**Published:** 2017-05-19

**Authors:** Hyun Ji Cho, Young Sang Kwon, Da‐Ran Kim, Gyeongjun Cho, Seong Won Hong, Dong‐Won Bae, Youn‐Sig Kwak

**Affiliations:** ^1^ Division of Applied Life Science (BK21 Plue) and IALS Gyeongsang National University Jinju Korea; ^2^ Environmental Chemistry Research Center Korea Institute of Toxicology Jinju Korea; ^3^ Department of Plant Medicine Gyeongsang National University Jinju Korea; ^4^ Center for Research Facilities Gyeongsang National University Jinju Korea

**Keywords:** antibiotic production, biological control, streptomycetes, *wblE2* transcription factors

## Abstract

*Streptomyces griseus* S4‐7 was originally isolated from the strawberry rhizosphere as a microbial agent responsible for Fusarium wilt suppressive soils. *S. griseus* S4‐7 shows specific and pronounced antifungal activity against *Fusarium oxysporum* f. sp. *fragariae*. In the *Streptomyces* genus, the *whi* transcription factors are regulators of sporulation, cell differentiation, septation, and secondary metabolites production. *wblE2* function as a regulator has emerged as a new group in *whi* transcription factors. In this study, we reveal the involvement of the *wblE2* transcription factor in the plant‐protection by *S. griseus* S4‐7. We generated Δ*wblE*, Δ*wblE2*, Δ*whiH*, and Δ*whmD* gene knock‐out mutants, which showed less antifungal activity both in vitro and *in planta*. Among the mutants, *wblE*2 mutant failed to protect the strawberry against the Fusarium wilt pathogen. Transcriptome analyses revealed major differences in the regulation of phenylalanine metabolism, polyketide and siderophore biosynthesis between the S4‐7 and the *wblE*2 mutant. The results contribute to our understanding of the role of streptomycetes *wblE2* genes in a natural disease suppressing system.

## 1 INTRODUCTION

Streptomycetes are Gram‐positive, mycelium‐forming, soil microorganisms that play important roles in nature. They are of great socio‐economic relevance because they produce many secondary metabolites that have been developed into clinical drugs (antibiotics, antitumorals, and immunosuppressants, amongst other) (Hopwood, 2007). Streptomycetes have a complex developmental cycle that resembles filamentous fungi, i.e., forming hyphae, mycelia, and spores. Traditional research of the streptomycetes developmental cycle mainly focused on sporulation phases in solid culture media (Flȁrdh & Buttner, 2009). After spore germination, viable vegetative (substrate) mycelium grows on the surface and inside the agar until it differentiates into a reproductive (aerial) mycelium that grows into the air, producing spores at the end of the cycle. Spore germination constitutes the first step of *Streptomyces* development (Kieser, Bibb, Buttner, Chater, & Hopwood, 2000).

The production of secondary metabolites by streptomycetes generally coincides with, or slightly precedes, the development of aerial hyphae in surface‐grown cultures (Guijarro, Suarez, Salas, & Hardisson, 1983). The secondary metabolite production and aerial hyphae formation presumably reflected the need to access multiple nutrients, optimize cellular morphology and metabolic differentiation (Chater & Horinouchi, 2003). These developmental stages govern pathway‐specific regulatory genes whose expression, in turn, frequently depends on genes required to produce secondary metabolites (Mikulik, Janda, Weiser, Stastná, & Jiranova, 1984). Some of these genes, notably the *whi* genes, are necessary for the full differentiation into mature spore chains (Bibb, 2005; Chater & Horinouchi, 2003; Elliot & Talbot, 2004). Remarkably, the w*hiB‐like* (*wbl*) family of regulators (named after the first discovered WhiB protein from *Streptomyces coelicolor*) is found only in Actinobacteria (Rybniker et al., 2010; Smith et al., 2010). Wbl proteins are generally small (10–15 kDa) and contain a highly‐conserved pattern of cysteine residues (Cys‐Xn‐Cys‐X2‐Cys‐X5‐Cys) that bind an iron–sulfur cluster (Jakimowicz et al., 2005). Cluster‐free Wbl proteins with disulfide bond play regulatory roles during developmental differentiation of streptomycetes (Crack et al., 2009). Impairment of the Wbl iron–sulfur cluster in bacteria affects Wbl disulfide bond reductase activity (Alam, Garg, & Agrawal, 2007; Beinert, Holm, & Munck, 1997; Kiley & Beinert, 2003). Among *wbl* regulators, *wblE2* is related to bacterial iron–sulfur cluster proteins that *wblE2* function as a regulator has emerged as a new group in *whi* transcription factors (Crack, Green, Hutchings, Thomson, & Le Brun, 2012). The transcription factors are known to involve from in oxidative stress, switching aerial hyphae formation, secondary metabolites production. The results of previous studies suggest that the *wbl* family of regulator governs various physiological and morphological changes in actinomycetes (Crack et al., 2012).

The *Streptomyces griseus* S4‐7 strain was isolated and characterized from a soil suppressing the strawberry Fusarium wilt disease caused by *Fusarium oxysporum* f. sp. *fragariae* (Cha et al., 2016). S4‐7 produces bioactive compounds that act to block fungal cell wall biogenesis and stability, resulting in the pathogen growth inhibition. The bioactive compounds and genome sequencing revealed not only a large number of genes dedicated to the secondary metabolite biosynthesis but also a high proportion and diversity of regulatory genes (Cha et al., 2016). In this study, the function of *wblE2* genes of the probiotic plant strain *S. griseus* S4‐7 was evaluated using mutagenesis and biological control experiments, as well as transcriptome analyses. The results contribute to our understanding of the role of streptomycetes *wblE2* genes in a natural disease suppressing system.

## 2 MATERIALS AND METHODS

### 2.1 Bacterial strains, plasmids, and culture conditions

Strains and plasmids used in this study are listed in Table 1. *S. griseus* S4‐7 was precultured in 25 ml of YEME medium (yeast extract‐malt extract: 3 g of yeast extract, 3 g of malt extract, 5 g of bacteriological peptone, 10 g of glucose, 170 g of sucrose per 1 L). S4‐7 and mutants were grown for 40 hr. The mycelium was harvested and washed twice with distilled water, then ground in 5 ml of 10.3% (w/v) sucrose with a glass bead homogenizer and sonicated with 30 pulses for 10 min to disrupt clumped mycelia. This approach allowed the bacterium development in an equivalent way (Salerno et al., 2013). Approximately 10^8^ colony forming units (cfu)/ml were inoculated onto nitrocellulose membranes coated the surface of the MS (mannitol soya flour agar: 20 g of mannitol, 20 g of soya flour, and 20 g of agar per 1 L) to obtain confluent growth. The mycelium was scraped from the cellophane discs at three different time points during colony development for further experiments

**Table 1:** List of strains and plasmids used in this study

Strains/plasmids	Description	Reference
*E. coli*		
DH5α	*F‐*,* Φ8dlacZ*ΔM15, (*lacZYA‐argF*)U169, *deoR*,* recA1*,* endA*1, *hsdR17* (rk‐,mk+), *phoA*,* supE44*, λ*‐*,* thi‐1*,* gyrA*96, *relA*1	Kieser et al. (2000)
ET12567/pUZ8002	*dam‐13*::Tn*9 dcm‐6 hsdM*; carries RK2 derivative with defective oriT for plasmid mobilization, Kan^r^	Kieser et al. (2000)
*S. griseus*		
S4‐7	Wild‐type	Cha et al. (2016)
Δ*wblE*2	S4‐7Δ*wblE*2::apr	This work
Δ*whiH*	S4‐7Δ*whiH*::apr	This work
Δ*whmD*	S4‐7Δ*whmD*::apr	This work
Δ*wblE*	S4‐7Δ*wblE*::apr	This work
S4‐7*wblE*2	S4‐7*wblE*2::hyg, *wblE2* complementation	This work
Plasmid		
pKC1132	*Streptomyces* suicide vector containing *oriT* RK2 for conjugative transfer from *E. coli* to *Streptomyces*	Bierman et al. (1992)
pIJ10257	Mobilizable vector, integrates at ΦC31 *attB* site, Hyg^r^	Novotna et al. (2012)
pKC46	pKC1132 containing *wblE*2 region	This work
pKC14	pKC1132 containing *whiH* region	This work
pKC54	pKC1132 containing *whmD* region	This work
pKC11	pKC1132 containing *wblE* region	This work
pIJ46	pIJ10257 containing *wblE*2 region	This work

### 2.2 Genome mining and homologous knockout mutant strains

*S. griseus* S4‐7 draft sequences were submitted to Rapid Annotation using Subsystem Technology (RAST Version 2.00). The RAST server is an automated service for the annotation pipeline (www.rast.nmpdr.org; Aziz et al., 2008). The *whi* families of regulators were mined based on functional annotation by RAST. To generate homologous deletion mutants, each gene was amplified by PCR as two fragments (LA and RA) with four primers (Table S1). Table S1 lists primer sequences and amplicon sizes. The PCR reaction volume was 40 μl, and each reaction contained 80 ng of S4‐7 genomic DNA, 10 pmol/L each primer, 200 μmol/L dNTP mixture, 20 μl of 2 × ToYoBo buffer, and 1 U KOD FX Neo polymerase (Toyobo, Japan). PCR was performed in a MJ Research PTC‐200 thermal cycler (Bio‐Rad, USA). An initial denaturation step at 98°C for 2 min was followed by 30 cycles consisting of denaturation at 98°C for 30 s, annealing at 55°C for 30 s, and extension at 72°C for 30 s. PCR amplicons were visualized by electrophoresis in 1.2% agarose gels. The bands were excised from the gels and eluted with a Dokdo‐Prep^™^Gel Extraction Kit (ElpisBiotech, Korea). To link the amplicons, PCR linkage of LA and RA partial gene fragments of each gene was performed. The link PCR volume was 20 μl, and it contained 2 μl of each purified LA and RA PCR product, 200 μmol/L dNTP mixture, 10 μl of 2 × ToYoBo buffer, and 1 U KOD FX Neo polymerase. Five cycles of PCR were performed (98°C for 30 s, 50°C for 30 s, and 72°C for 30 s) as the first round of linkage PCR. After the first round, a second round of PCR was carried out (30 cycles of 98°C for 30 s, 55°C for 30 s, and 72°C for 35 s). The LA:RA linkage was purified by gel elution and cloned into a pGEM‐T easy vector with Rapid T4 DNA ligase (Promega, USA). The cloned construct in pGEM‐T Easy was verified by sequencing. The LA:RA linkage fragment was isolated after digestion with *Eco*RV and *Bam*HI, and cloned into the same sites of pKC1132 using T4 DNA ligase. The construct was used to transform *Escherichia coli* ET12567 containing pUZ8002. A single colony of *E. coli* ET12567/pUZ8002 carrying an *ori*T was inoculated into 10 ml of LB containing 25 μg/ml chloramphenicol (Cm; to select for *dam*::Tn*3*), 25 μg/ml kanamycin (Km; to select for pUZ8002), and an appropriate antibiotic for the selection of the *ori*T‐containing vector. The culture was incubated at 37°C overnight, then diluted 1:100 in fresh LB containing the antibiotics, and grown at 37°C until OD_600_ = 0.4–0.6. *E. coli* cells were then washed twice with an equal volume of antibiotic‐free LB and resuspended in 0.1 volume of LB. For each conjugation reaction, 0.5 ml of *E. coli* cells was required. For each conjugation, 10^8^ cfu/ml *S. griseus* S4‐7 spores (spore was collected from MS media grown colonies and mixed by gentle pipetting 20% glycerol as final concentration then the stock stored at −80°C) were added to 500 μl of 2 × YT (16 g of Bacto tryptone, 10 g of Bacto yeast extract, and 5 g of NaCl per 1 L), incubated at 50°C for 10 min to induce germination, then cooled to room temperature. *E. coli* cells (0.5 ml) were added to 0.5 ml of heat‐shocked S4‐7 spores. The mixture was centrifuged for a few seconds (a pulse), the supernatant was discarded, and the cell pellet was resuspended in the residual liquid (~100 μl). The suspension was spread on a MS agar plate supplemented with 10 mmol/L MgCl_2_, which had been dried in a laminar flow cabinet for 1 hr prior to plating. After incubation at 28°C for 16–20 hr, the plate was overlaid with 0.5 ml of distilled water containing 25 μl of nalidixic acid (Ndx; final concentration 20 μg/ml, assuming 25 ml of agar per plate), to eliminate the *E. coli* donor, and the appropriate antibiotic for plasmid selection. The overlay was distributed evenly using a glass spreader. The plate was left in a laminar flow cabinet until all the liquid had been absorbed. Incubation was then continued at 28°C.

### 2.3 Overexpression of the *wblE2* gene

To overexpress the function of the WhiB‐type transcriptional regulator (*wblE2*) in the *S. griseus* S4‐7, the gene was cloned into pIJ10257 (Novotna, Hill, Vincent, Liu, & Hong, 2012) under the control of the ermE* promoter (*ermEp*). The gene was first amplified by PCR using primers C.46.F (CATATGTTGAGCAGCACCGAGAAC) and C.46.R (TTAATTAATTGCCGGATCACGAACAC). The PCR reaction volume was 40 μl, and each reaction contained 80 ng of S4‐7 genomic DNA, 10 pmol each primer, 200 μmol/L dNTP mixture, 20 μl of 2 × ToYoBo buffer, and 1 U KOD FX Neo polymerase (Toyobo, Japan). PCR was performed in a MJ Research PTC‐200 thermal cycler (Bio‐Rad, USA). An initial denaturation step at 98°C for 2 min was followed by 30 cycles consisting of denaturation at 98°C for 30 s, annealing at 58°C for 30 s, and extension at 72°C for 30 s.

The cloning and conjugation protocols were followed as described above. For transformant selection, following incubation for 6 hr at 30°C, 500 μl of antibiotic solution [20 μl of Ndx (stock solution, 25 mg/ml) and 80 μg/ml hygromycin] was spread on the conjugation plate and air‐dried for 10–20 min in a sterile hood. Plates were incubated for 3 days at 30°C. Bacterial genomic DNA was purified using a Solg^™^ genomic DNA PrepKit (Solgent LTD, Korea), and PCR was performed with hygromycin detection primers Hyg‐det5 (CGGCTCATCACCAGGTAGGG) and Hyg‐det3 (TCCGCTGTGACACAAGAATC) to verify the integration of pIJ10257 into the *S. griseus* S4‐7 genome. Overexpressed *S. griseus* S4‐7 colonies were randomly picked and checked for their ability to inhibit *F. oxysporum* growth.

### 2.4 In vitro and in planta antagonistic and biocontrol activity assays

Loss of function of the member of the *whi* type transcription regulators in S4‐7 mutants were assessed by evaluating their activity against the Fusarium wilt pathogen both in vitro and *in planta*. Fungal discs (5 mm diameter) with cultures pre‐grown for 7 days on PDA at 27°C were placed at the center of PDK plates (24 g of potato dextrose, 10 g of peptone, and 18 g of agar per 1 L). The wild‐type and mutant strains, pre‐grown for 5 days on TSB at 28°C, were transferred to the opposite sides of plates, 2.5 cm away from the fungal disc. PDK plates were then incubated for 7 days at 28°C. Inhibition zones were measured every day, and the antifungal activity of the wild‐type and mutants was evaluated after 7 days.

To evaluate the biological control activity of the *whi*‐type transcription regulators, S4‐7 and mutant strains were grown in PDK broth for 7 days at 28°C, following which the cell densities were adjusted to 4.2 × 10^7^ cfu/ml. Strawberry roots were dipped in bacterial suspensions for 5 min. The following seven treatments were evaluated: CK, control with 0.1% methylcellulose; S, *S. griseus* S4‐7 with 0.1% methylcellulose; F9, *F. oxysporum* chlamydospores (1.6 × 10^5^ cfu/g of soil); Δ*wblE*2, Δ*wblE*2 with 0.1% methylcellulose; Δ*wblE*, Δ*wblE* with 0.1% methylcellulose; Δ*whmD*, Δ*whmD* with 0.1% methylcellulose; and Δ*whiH*, Δ*whiH* with 0.1% methylcellulose. *F. oxysporum* chlamydospore inoculum was added to each soil sample at a dose of 1.6 × 10^5^ cfu/g of soil, except control treatment. The soil was added to plastic pots (22 × 60 × 18 cm), and 50‐d‐old strawberry (cv. Sulhyang) plants were planted in each pot. The treatments were arranged in a complete randomized block design and replicated five times. Plants were incubated in a growth chamber (25°C; 16 hr light/8 hr dark cycle), and the incidence and severity of disease were observed every 7 days up to 45 days after planting. The incidence and severity of the disease were evaluated using a 0–5 scale, every week: 0, healthy; 1, 1 to 3 leaves rolled and yellowed; 2, 3 to 4 leaves rolled and deformed; 3, chlorosis and early plant wilting; 4, necrosis and entire plant wilting; and 5, dead or nearly so. The results were statistically analyzed by Tukey's HSD test (with significance set at *p *=* *.05) using SigmaPlot ver. 11.0 (Systat software INC., CA, USA).

### 2.5 Transcriptome analysis and data mining

Total RNA was extracted from cells at three different time points (24 hr, 48 hr, and 72 hr) using TRizol (van Dessel, van Mellaert, Geukens, Lammertyn, & Anné, 2004). The quantity and quality of total RNA were evaluated using RNA electrophoregrams with an Agilent 2100 Bioanalyzer (Agilent Technologies, Germany) and by assessing the RNA integrity number. Total RNA (5.5 μg) was treated with a MICROBExpress^™^ mRNA Enrichment Kit (Ambion, Foster City, CA, USA). The resulting mRNA samples were processed into sequencing libraries using an Illumina mRNA‐Seq Sample Preparation Kit (Illumina, San Diego, CA, USA) following the manufacturer's protocols. One lane per sample was used for sequencing on an Illumina Genome Analyzer IIx (Illumina, CA, USA) to generate nondirectional, single‐ended, 36 bp reads. Quality‐filtered reads were mapped to reference genome sequences using CLRNASeq version 1.00 (Chunlab, Seoul, Korea).

Data mining was performed using CLRNASeq version 1.00 (Chunlab, Seoul, Korea). *S. griseus* S4‐7 genes were sorted during Differentially Expressed Gene (DEG) analysis, after normalization with Trimmed Mean of M‐values (TMM) (Robinson & Oshlack, 2010). The following DEG analysis parameters were used: cut‐off fold‐change >5 and *p*‐values of Empirical Analysis of Digital Gene Expression Data in R (edgeR) ≤0.005 difference between the mutant and the wild type. The sorted genes were classified into evolutionary genealogy of genes with Non‐supervised Orthologous Groups (eggNOG) categories. The results were visualized by gplots version 3.0.1 and ggplot2 version 2.1.0 of the R package. Differential expressed genes between the mutant and the wild type were also analyzed, using KEGG pathways to assign the biological function of the genes.

### 2.6 Validation of transcriptome analyses with qPCR

RNAs were extracted from the wild type and mutant as described above, and cDNAs were synthesized using a ReverTra Ace‐α‐^®^ kit, following the manufacturer's protocol (ToYoBo, Japan). Each sample (20 μL) included 500–600 ng of RNA, 5 × RT buffer (4 μl), dNTP mixture (2 μl), random primer (1 μl), RNase inhibitor (1 μl), ReverTra Ace (1 μl), and RNase‐free water. The reverse transcription reaction was performed at 30°C for 10 min and 42°C for 20 min, heated up to 99°C for 5 min, and then cooled to 4°C. The list of validated genes is presented in Table S2. Validating PCR analyses were carried out with the CFX connect Real‐Time PCR Detection System (BioRad, CA, USA), and the reaction mixtures contained 2 μl of 5‐fold diluted cDNA, 10 pmol each primer, 10 μl of Universal SYBR^®^ Green Supermix (BioRad, CA, USA), and 4 μl of sterilized water. After an initial denaturation at 95°C for 30 s, the following PCR steps were repeated 40 times: 95°C for 15 s, 60°C for 15 s, and 72°C for 30 s. Melting curves were generated by heating at 95°C for 10 s, followed by cooling to 65°C, and slowly heating the samples at 0.5°C /s to 95°C.

## 3 RESULTS

### 3.1 *S. griseus* S4‐7 *whi* genes and mutant phenotypes

**Figure 1:**
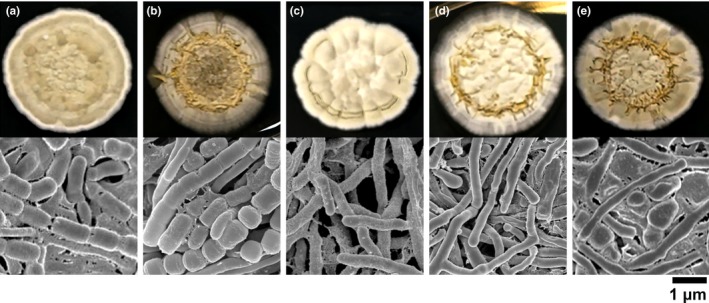
Colony morphology of member of *whi* transcription factor mutants. Cultures of the mutants were incubated for 7 days on PDK medium at 30°C. (a) wild‐type (S4‐7); (b) Δ*wblE*; (c) Δ*whiH*; (d) Δ*whmD*; E, Δ*wblE*2

The genomic sequence of *S. griseus* S4‐7 has been deposited (GenBank Accession: GCA_000932225.1) and analyzed in RAST server (http://rast.nmpdr.org). Genes encoding ten members of *whi* transcription factors or related genes have been identified in the S4‐7 genome based on functional annotation by RAST (Table S1). To further reveal the function of these genes, we obtained four mutant stains, following Δ*wblE*, Δ*wblE2*, Δ*whiH*, and Δ*whmD* (Figure S1). Their functions in streptomycetes have not been clearly verified, except for the *whiH* gene. *whiH* participates cell division in sporulation stage (McCormick & Fl__rdh, 2012). The loci were disrupted by homologous knockout mutagenesis using pKC1132 suicide vector system. The four mutants showed reduced differentiation and aerial mycelium/spore formation compared with the wild type (Table 2 and Figure 1). The color of the wild‐type aerial mycelium was gray because of spore development, while the mutant mycelia were yellow‐white. The Δ*wblE* and Δ*wblE*2 strains showed similar phenotypical characteristics to S4‐7 at the early stage of aerial mycelium formation. However, Δ*whiH* and Δ*whmD* were significantly less gray and spore development (Figure 1). The result suggests that *wblE* and *wblE2* gene may less involve cell development and sporulation in *S. griseus* S4‐7.

**Table 2:** Sporulation phenotype, and antifungal and biocontrol activities of *whi* mutants

Strain	Gene function	Sporulation	in vitro antifungal activity	*in planta* plant protection
Δ*wblE*	*whiB*‐like transcription regulator	+	‐	‐
Δ*whiH*	Transcriptional regulator, *GntR* family	++	+	++
Δ*whmD*	*whiB*‐type transcriptional regulator	+	‐	+
Δ*wblE*2	*whiB*‐type transcriptional regulator	+	‐	‐
S4‐7	Wild‐type	++++	++++	++++

### 3.2 Plant protection ability of member of the *whi* transcription family

**Figure 2:**
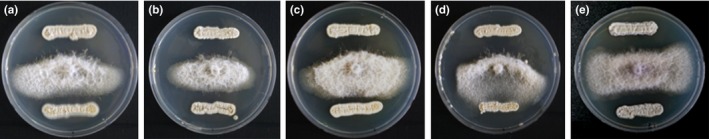
Antagonism test of w*hi* mutants. The mutants (a, Δ*wblE*; b, Δ*whiH*; c, Δ*whmD*; d, Δ*wblE2,* E: overexpression of *wblE*2 gene) lost their antagonistic activity against *F. oxysporum*, as assayed on PDK medium at 28°C for 7 days. The wild‐type, S4‐7, was streaked on the top of each plate

The antifungal activity and plant protection ability of the mutants were determined both *in vitro* and *in planta*. In vitro, the antifungal activity against the wilt disease pathogen of Δ*wblE*, Δ*whmD*, and Δ*wblE*2 strains was decreased in comparison with the wild type (Figure 2). The Δ*whiH* antifungal activity was not diminished compared with that of the wild type (Figure 2). This was especially evident for the Δ*wblE*2 mutants, which lost their antifungal activity completely. To verify *wblE*2 gene function, a *wblE*2 overexpression strain was constructed, where a single copy of *wblE2* was expressed under a strong constitutive *ermE* promoter. The overexpression *wblE*2 strain fully suppressed *F. oxysporum* (Figure 2).

**Figure 3:**
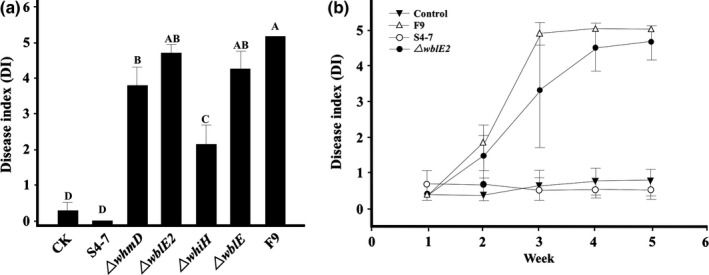
Biological control ability of *S*. *griseus* S4‐7 and mutants in growth chamber experiments. (a) CK, control; S, *S. griseus* S4‐7 with 0.1% methylcellulose; F9, *F. oxysporum* chlamydospores (1.6 × 10^5^ cfu/g of soil), each mutant (4.2 × 10^7^ cfu/ml) was treated with 0.1% methylcellulose. *F. oxysporum* chlamydospore inoculum was added to the soil samples at a dose of 1.6 × 10^5^ cfu/g of soil, following which the strawberries (50‐d‐old cv. Sulhyang) were planted in each pot. B; Disease incidence and severity were assessed over 45 days after planting. Disease index represents the number of strawberry leaves with symptoms: 0, healthy; 1, 1 to 3 leaves rolled and yellowed; 2, >3 leaves rolled and deformed; 3, chlorosis and early plant wilting; 4, necrosis and entire plant wilting; and 5, dead or nearly dead

Regarding the mutants’ disease suppressive activity *in planta*, Δ*wblE*2, Δ*whmD*, Δ*wblE*, and Δ*whiH* mutants failed to fully protect the strawberry against the Fusarium wilt pathogen (Figure 3 and Figure S2). The disease progression and severity of Δ*wblE*2‐assisted plants were similar to those of pathogen‐only treated plants (Figure 3b). The mutant significantly differed from the *S. griseus* S4‐7 with respect antifungal/plant protection ability. The results indicated that *wblE*2 transcription factor may be a responsible factor of *S. griseus* S4‐7 in suppressing the wilt disease occurrence.

### 3.3 Transcriptome of the Δ*wblE*2 mutant

To begin to reveal the difference between S4‐7 and Δ*wblE*2 mutant in secondary metabolite production, we generated six RNA samples from the both strains, at different time points (24, 48, and 72 hr). RNA was isolated from three independent cultures of each strain at each time point. The obtained total RNA quantities suggested a reliable concentration, volume, amount, rRNA ratio, and RNA integrity number (RIN) value. Expressed sequence tag or cDNA sequencing allow identification of highly expressed transcripts. Here, cDNA libraries were generated and subjected to massively parallel sequencing using Illumina technology, and used to define exons, 5′ and 3′ boundaries, and introns, as well as to quantify gene expression levels. For each time point, cDNAs of the wild type and the Δ*wblE2* strain were mapped to the S4‐7 reference genome. In total, 7,501 genes were detected as expressed in six different class comparisons. When only considering the differences in expression with trimmed mean of M (TMM) values fold‐change >2 and by edgeR (*p *<* *.005) as significant, we identified 414 genes differentially expressed in S4‐7 and the mutant (Table S3). Among the differently expressed genes, only 33 were significantly up‐regulated in the Δ*wblE*2 strain compared with the wild type (Table S3 and Figure S3). By contrast, 381 genes were dramatically down‐regulated in the Δ*wblE*2 strain. Based on GO functional assignment, carbohydrate transport and metabolism (40 genes), transcription (32 genes), inorganic ion transport and metabolism (20 genes), amino acid transport and metabolism (15 genes), signal transduction (15 genes), and energy production and conversion (10 genes) were differently expressed in the Δ*wblE*2 strain (Figure S3).

**Figure 4:**
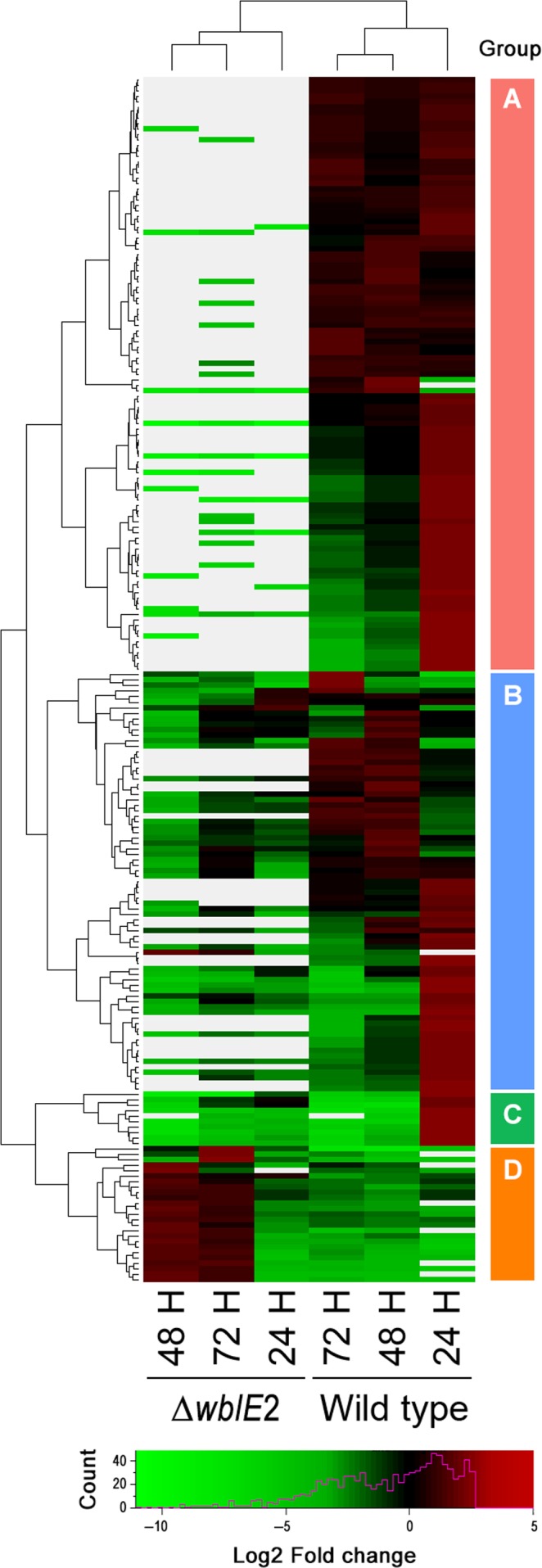
Heatmap of mutant and wild‐type RNA‐seq transcriptomes. The DEG cut‐off was set at Fold‐change >5 and *p *≤* *.005 in edgeR. Sorted genes were classified with eggNOG and visualized by gplots version 3.0.1 and ggplot2 version 2.1.0 (R package)

The differently expressed genes were categorized into four groups based on their expression patterns (Table 3 and Figure 4): group A, not expressed in Δ*wblE2* regardless of the time point; group B, a relative decrease in expression in the Δ*wblE2* strain depending on the time point; group C, similar expression patterns between the wild type and the mutant except for the 24 hr; and group D, higher expression in Δ*wblE2* than in the wild‐type strain. Gene functions in each group were assigned to biological pathways by the KEGG database. Group A could be further separated into two subgroups based on the biological function of the genes. The first subgroup was related to bacterial volatile metabolite metabolism, and the genes were significantly down‐regulated in the Δ*wblE2* strain. The expression of genes involved in polycyclic aromatic hydrocarbon degradation, chloroalkane degradation, degradation of aromatic compounds, and ethylbenzene degradation was significantly lower in the Δ*wblE2* strain than in the wild‐type strain. The functions of genes in the second subgroup were associated with antibiotic and metabolite biosynthesis pathways, phenylalanine metabolism, and polyketide biosynthesis, and their expression was also significantly suppressed in the mutant. In group B, the differently expressed genes were mainly related to antibiotic and nutrient metabolism. Sulfur metabolism, polyketide biosynthesis, siderophore biosynthesis, sugar metabolism, and amino acid metabolism were all down‐regulated in the Δ*wblE2* strain compared with the S4‐7 wild‐type. The cytochrome P450 gene was only highly expressed in the vegetative hyphae stage of the wild‐type strain and belonged to group C. In group D, two ABC transporters were detected as up‐regulated in the mutant strain. Validation of transcriptome results by real‐time PCR verified that the expression of antibiotic‐related genes was significantly reduced in the Δ*wblE2* strain compared with the wild type (Figure S4). The expression of genes related to phenylalanine biosynthesis and bisphenol degradation was completely shut down in the mutant (Table S3). Transcriptome data presented that majority of the down regulated genes in Δ*wblE2* strain are related antibiotic precursor or synthesis pathway.

**Table 3:** Genes identified as differentially regulated by transcriptome comparisons of *S. griseus* S4‐7 and Δ*wblE*2 mutant and their biological pathways

Gene name	KEGG pathway
Group A	Not expressed in *wblE2* mutant
SGS47207490	Two‐component system
SGS47207500	Two‐component system
SGS47208000	Selenocompound metabolism; Stilbenoid, diarylheptanoid, and gingerol biosynthesis; Polycyclic aromatic hydrocarbon degradation
SGS47208360	Limonene and pinene degradation; Stilbenoid, diarylheptanoid, and gingerol biosynthesis; Aminobenzoate degradation; Polycyclic aromatic hydrocarbon degradation
SGS47208040	Nucleotide excision repair
SGS47208350	Carotenoid biosynthesis
SGS47208060	Fructose and mannose metabolism; Butanoate metabolism; Linoleic acid metabolism; Chloroalkane and chloroalkene degradation; Bisphenol degradation
SGS47208030	ABC transporters
SGS47208230	Phenylalanine metabolism
SGS47208250	Degradation of aromatic compounds; Tyrosine metabolism
SGS47208070	Fructose and mannose metabolism; Amino sugar and nucleotide sugar metabolism
SGS47208140	Phenylalanine, tyrosine, and tryptophan biosynthesis
SGS47208130	Phenylalanine, tyrosine, and tryptophan biosynthesis
SGS47207970	Phosphotransferase system (PTS)
SGS47208170	Tyrosine metabolism; Limonene and pinene degradation; Benzoate degradation; Aminobenzoate degradation; Ethylbenzene degradation
SGS47208210	Polyketide sugar unit biosynthesis; Biosynthesis of vancomycin group antibiotics; Streptomycin biosynthesis
SGS47208110	Polyketide sugar unit biosynthesis
SGS47208090	Amino sugar and nucleotide sugar metabolism; Two‐component system
SGS47188580	Salivary secretion
SGS47208490	Steroid degradation
SGS47208530	Histidine metabolism; Limonene and pinene degradation; Aminobenzoate degradation; Chlorocyclohexane and chlorobenzene degradation; Toluene degradation; Bisphenol degradation; Naphthalene degradation; Polycyclic aromatic hydrocarbon degradation
SGS47208370	Steroid degradation
SGS47209050	Salmonella infection
Group B	Down‐regulated in *wblE2* mutant
SGS47149320	Sulfur metabolism; Purine metabolism; Selenocompound metabolism
SGS47207010	Starch and sucrose metabolism
SGS47184540	Lysine degradation
SGS47196940	ABC transporters
SGS47207550	Glyoxylate and dicarboxylate metabolism; Nitrogen metabolism
SGS47207540	Butanoate metabolism; C5‐Branched dibasic acid metabolism; Valine, leucine, and isoleucine biosynthesis; Pantothenate and CoA biosynthesis
SGS47142800	Complement and coagulation cascades
SGS47199650	Nicotinate and nicotinamide metabolism
SGS47199660	Microbial metabolism in diverse environments
SGS47208180	Polyketide sugar unit biosynthesis; Streptomycin biosynthesis
SGS47190590	FoxO signaling pathway; cGMP‐PKG signaling pathway; AMPK signaling pathway; Insulin signaling pathway; Adipocytokine signaling pathway; Adipocytokine signaling pathway; Type II diabetes mellitus
SGS47208430	Biosynthesis of siderophore group nonribosomal peptides
SGS47208400	Porphyrin and chlorophyll metabolism
SGS47145360	Amino sugar and nucleotide sugar metabolism
SGS47208310	Histidine metabolism
SGS47208460	Arginine and proline metabolism
SGS47208320	d‐Alanine metabolism; *Staphylococcus aureus* infection
SGS47208120	Metabolism of xenobiotics by cytochrome P450; Bile secretion; Chemical carcinogenesis
SGS47208290	Biosynthesis of siderophore group nonribosomal peptides
Group C	Equally expressed
SGS47146480	Pentose and glucuronate interconversions; Metabolism of xenobiotics by cytochrome P450
Group D	Overexpressed in *wblE2* mutant
SGS47190030	ABC transporters
SGS47190040	ABC transporters

## 4 DISCUSSION

The genus *Streptomyces* is considered a source of various secondary metabolites, especially antibiotics. Nearly 70% of clinical antibiotics were developed from the products of this genus (Bibb, 2013). However, the knowledge of the role of *Streptomyces* in microbe‐plant interactions and in agriculture is relatively limited. *S. griseus* S4‐7 was isolated from the strawberry rhizosphere in a 15‐year continuous monoculture field highly suppressive to Fusarium wilt disease (Cha et al., 2016). This strain exerts its antifungal effect by disturbing fungal cell wall remodeling and RNA polymerase activity (Cha et al., 2016). Different secondary metabolic gene clusters respond to various environmental and physiological signals and stress conditions (Bibb, 2005, 2013). Moreover, activation of a secondary metabolism gene cluster is usually correlated with morphological development, from surface‐grown to sporulation, a process that is subject to its own regulatory checkpoints and complex signaling cascades. Spores of *S. griseus* S4‐7 germinate, and the germ tube elongates into long branching hyphae that grow into the substrate and form vegetative mycelium. Upon differentiation, aerial mycelium is formed, which then develops into chains of spores (Soliveri, Gomez, Bishai, & Chater, 2000). Cell differentiation is triggered by nutrient depletion and other signals, and both the production of secondary metabolites and morphological differentiation are initiated. *whi* mutants defective in different sporulation stages are employed in genetic and biochemical analyses of these developmental stages (Yagüe, López‐Garcia, Rioseras, Sánchez, & Manteca, 2013). The regulation of secondary metabolism in streptomycetes is diverse and complex. Antibiotic production and antimicrobial activity observed in the laboratory are coordinated with the morphological development in surface‐grown cultures (Bibb, 2005). Developmental regulatory genes, members of the *wbl* transcription factor, are only found in Actinobacteria (Soliveri et al., 2000). Several genes responsible for white (*whi*) or light gray colonies with aerial hyphae are related to the production of gray spore pigment and cell arrest at different developmental stages. However, *wblE*, and *wblE*2 transcription factors have no assigned function in *Streptomyces* spp. In this study, we present four Δ*wblE*, Δ*wblE2*, Δ*whiH*, and Δ*whmD* mutants. However, other six *whi* genes mutant could not be generated in our laboratory currently. From the mutants examined, Δ*whiE* and Δ*whiE2* failed to protect the strawberry against *F. oxysporum*. The *whiE2* gene overexpressed strain had the same antifungal activity as the wild type. This confirmed the physiological importance of *wbl* clusters as regulators that have evolved to enable specific physiological changes. The data indicated that the *wblE2* gene regulates the production of secondary metabolites that strongly contribute to the strawberry‐protecting property of S4‐7.

From 7,501 expressed genes, 5.5% (414 genes) were detected as differently regulated in the Δ*wblE2* mutant. The differently expressed genes were divided into four groups based on their expression patterns and expression intensities. Genes in group A, only weakly expressed in the mutant, suggested that the Δ*wblE*2 mutant may produce fewer antibiotic‐related secondary metabolites but with increased bacterial volatile emission compared with the wild type. A reduction of antibiotic production after loss‐of‐function mutation of *whi* transcription factors in *Streptomyces* has been well‐documented in previous studies (Bibb, 2005, 2013; Burian et al., 2012; Rybniker et al., 2010). However, the involvement or regulatory role of *wblE2* transcription factor in secondary metabolite and antibiotic production was not described. Transcriptome analysis revealed that sulfur metabolism and expression of related genes were correlated with antifungal activity in S4‐7. However, the Δ*wblE2* mutant had defective sulfur metabolism. In this mutant, not only was sulfur metabolism decreased but also polyketide, siderophore. The data clearly indicated that the *wblE2* transcription factor governs and contributes to specific metabolite production and activities of *S. griseus* S4‐7, including protecting the strawberry against the Fusarium wilt pathogen. Conprimycin (a novel thiopeptide) was highlighted in our previous study as the key antifungal polypeptide of the suppressive system (Cha et al., 2016). However, the expression levels of conprimycin or other thiopeptide biosynthesis genes in both the Δ*wblE2* mutant and the wild type were similar (data not shown). This result suggests two possible reasons, the first is that S4‐7 cells produce several antibiotics and each pathway may have an independent regulatory system. And second is that the conprimycin, which ribosomally synthesized a small peptide, may regulate post‐translational modification to be activation, such as cleavage prepeptide, azoline formation, dehydration of Ser/Thr residues, i.e. (Zheng, Fang, & Liu, 2017).

In conclusion, the plant probiotic strain, *S. griseus* S4‐7, protects the strawberry against Fusarium wilt disease and *wblE2* transcription factor is extensively involved in the regulation of antifungal secondary metabolites in the strain. The identified *wblE2* transcription factor regulates antibiotic, polyketide, xenobiotic, and siderophore metabolites in *S. griseus* S4‐7. The *wblE2* transcription factor plays the role of a master regulator in antifungal metabolite production in *S. griseus* S4‐7. Further studies of *S. griseus* S4‐7 will enable the development of better‐integrated models of *Streptomyces* responses to particular conditions and the involvement of this transcription factor in plant protection phenomena.

## CONFLICT OF INTEREST

None declared.
