# Supplementary material for: wblE2 transcription factor in Streptomyces griseus S4‐7 plays an important role in plant protection
Source: Microbiologyopen. 2017 May 19;6(5):e00494. doi: 10.1002/mbo3.494 (PMC5635160; doi:10.1002/mbo3.494)

**SUPPLEMENTAL INFORMATION**

Table S1. List of *whi* genes in the *S. griseus* S4-7 genome and details of primers used to generate homologous knockout mutants

| Gene name | Gene  function | Primer sequences (5′ to 3′) | | Amplicon size (bp) |
| --- | --- | --- | --- | --- |
| *wblE2* | WhiB-type transcriptional regulator | LA F^2^ | GATATCTTGAGGCGCGTCGATCGTC | 87 |
|  |  | LA R | AAGCTTGGTACCGAATTCACTCTGCTCACTCCCGAA |  |
|  |  | RA F | GAATTCGGTACCAAGCTTTGTTCTTCCCCATCGGGA | 137 |
|  |  | RA R | GGATCCCGCCCCAGATACCGGAGTC |  |
| *whiB* | Sporulation regulatory protein WhiB | LA F | GATATCTTCCTCGGCATGTCCTCGGCA | 273 |
|  |  | LA R | AAGCTTGGTACCGAATTCTTTCGGGCATTGGGTTGA |  |
|  |  | RA F | GAATTCGGTACCAAGCTTATTTTTGCTGATTTGAAC | 282 |
|  |  | RA R | GGATCCTTCTCGGGGAAGAAGGAC |  |
| *whmD* | WhiB-type transcriptional regulator | LA F | GATATCGATTGATGAGTCGTTGATC | 142 |
|  |  | LA R | AAGCTTGGTACCGAATTCGGCCGGGAAGAAGAACTC |  |
|  |  | RA F | GGATCCGAACGCCCCAGGGGTTTA | 97 |
|  |  | RA R | GGATCCAGGTGTCGAAGAAACGTTTC |  |
| *wblC* | WhiB family transcriptional regulator | LA F | GATATCACCCACCACAGGAGCAGA | 180 |
|  |  | LA R | AAGCTTGGTACCGGATTCATGTGCACTAGGTCGTAG |  |
|  |  | RA F | GAATTCGGTACCAAGCTT GAGGTCTTCTTCGCCGAG | 267 |
|  |  | RA R | GGATCCGACGGTCGATCGTTCCGA |  |
| *wblE* | WhiB-like transcription regulator | LA F | GATATCTGGAAGGGCCGAAAGTCC | 285 |
|  |  | LA R | AAGCTTGGTACCGAATTCGCTTGTGAATGTGAACGC |  |
|  |  | RA F | GAATTCGGTACCAAGCTTTTTGTCGTGAGGAAGACC | 171 |
|  |  | RA R | GGATCC CGCTGAGGCCACCCCAGA |  |
| *wblA* | WhiB-type transcription regulator | LA F | GATATCGTGATGAACGTGTCGCAG | 381 |
|  |  | LA R | AAGCTTGGTACCGAATTCCCATGAAAAAGGGACAGTG |  |
|  |  | RA F | GAATTCGGTACCAAGCTTATGAACTGTTCGTACAAG | 282 |
|  |  | RA R | GGATCCGCGTACGTCTCGTGCAGTTC |  |
| *wblB* | Sporulation regulatory protein WhiD | LA F | GATATCTCGAAAGAGTTACGGGTC | 330 |
|  |  | LA R | AAGCTTGGTACCGAATTCCGGGAGAAATCTGCCATTG |  |
|  |  | RA F | GAATTCGGTACCAAGCTTACCTCCGCGAAGGAGGTG | 249 |
|  |  | RA R | GGATCCAGGTGTCGAAGAAACGTTTC |  |
| *whiG* | RNA polymerase sigma factor SigB | LA F | GATATCGTGAAAAGGTAAGGCCGA | 339 |
|  |  | LA R | AAGCTTGGTACCGAATTCGTAGGAACGCCACAACTC |  |
|  |  | RA F | GAATTCGGTACCAAGCTTCACGCCGTTTTCAGCCAG | 231 |
|  |  | RA R | GGATCCCTCGTAGTAGTAGAGGGTC |  |
| *whiH* | Transcriptional regulator, GntR family | LA F | GATATCGACCGCTATCCCTATCCG | 171 |
|  |  | LA R | AAGCTTGGTACCGAATTCGTCACCGGAAACGATCATC |  |
|  |  | RA F | GAATTCGGTACCAAGCTTGACTGGAACCTGCTGGAT | 288 |
|  |  | RA R | GCGGCCGCGGAAAGGTGTTCGAGCAT |  |
| N/A^1^ | WhiB-type transcriptional regulator | LA F | GATATCATACGGCACCGCGGACGA | 255 |
|  |  | LA R | AAGCTTGGTACCGAATTCATGCCAGGGACCCGCATC |  |
|  |  | RA F | GAATTCGGTACCAAGCTTTCATGATCGAGTGCCGGG | 177 |
|  |  | RA R | GGATCCCTTCGCGTACGCGTTCAG |  |

^1^ The gene name was not annotated by RAST.

^2^ LA, left arm; RA, right arm; F, forward; R, reverse.

Table S2. Real-time PCR primers and their target genes

| Primer | Gene ID | Gene function | Sequence (5′ to 3′) |
| --- | --- | --- | --- |
| Sugar syn qF | SGS47208210 | Streptomycin synthesis, polyketide sugar synthesis | CGGTTTCATCGGCTCGCAGTTC |
| Sugar syn qR |  |  | GAGTACGTCAGCTTGTCCAGGACC |
| Str syn qF | SGS47208070 | Streptomycin synthesis | CGGTCTGCGCCTCACCTACATA |
| Str syn qR |  |  | CAGGTCTCCGTCCAGCATGATG |
| Xeno qF | SGS47208120 | Xenobiotics | GAGTGCCGCTGTCCTACCTCAA |
| Xeno qR |  |  | TCGTGGTGACGAACTGGGGGAA |
| Ion sulfur qF | SGS47199660 | Sulfur metabolism | CGACAAGGGAGGCTCCGACAC |
| Ion sulfur qR |  |  | GGGTCACCACGACTTTCTGTTGC |
| Tyr bio syn qF | SGS47208140 | Phenylalanine, tyrosine, and tryptophan biosynthesis | TGGTCTGATGGCTCACCACCC |
| Tyr bio syn qF |  |  | TCGGTGGAGTTGAGATGCGAGA |
| Amino deg qF | SGS47208360 | Bisphenol degradation, aminobenzoate degradation | CCAGGACGCCAGGAGCTACG |
| Amino deg qR |  |  | CGTGATCGGGTGGGTGCTGTA |
| Tyr metabol q | SGS47208250 | Tyrosine metabolism | CGCCGACTTCTACGAGCCCTTC |
| Tyr metabol qR |  |  | CGTGGTTCCAGTACAGCACCTTCTC |
| Sigma ECF qF | SGS47209230 | RNA polymerase sigma-70 factor, ECF subfamily | CCCGCTGATCGACGAAGCC |
| Sigma ECF qR |  |  | TGGAGGTAGGTCATCACCATGACGT |
| RecA qF | recA | Housekeeping gene | CGCTCGCACAGATTGAACGACAG |
| RecA qR |  |  | GGTCCGTACACCTCCACCACG |
| hrdA F | hrdA | Housekeeping gene | GCTGACCATGCTCGATCTC |
| hrdA R |  |  | GGCGTACGTGGAGAACTTGT t |

Fig S1. Position of homologues knock-out of *whi* gene clusters in *S. griseus* S4-7. The genes and their predicted products are as follows: A, *wblE*; B, *wblE*2; C, *whiH*; D, *whmD*. The prediction and annotation were performed using RAST server (rast.nmpdr.org). Black triangles indicate the position of knocked-out genes.


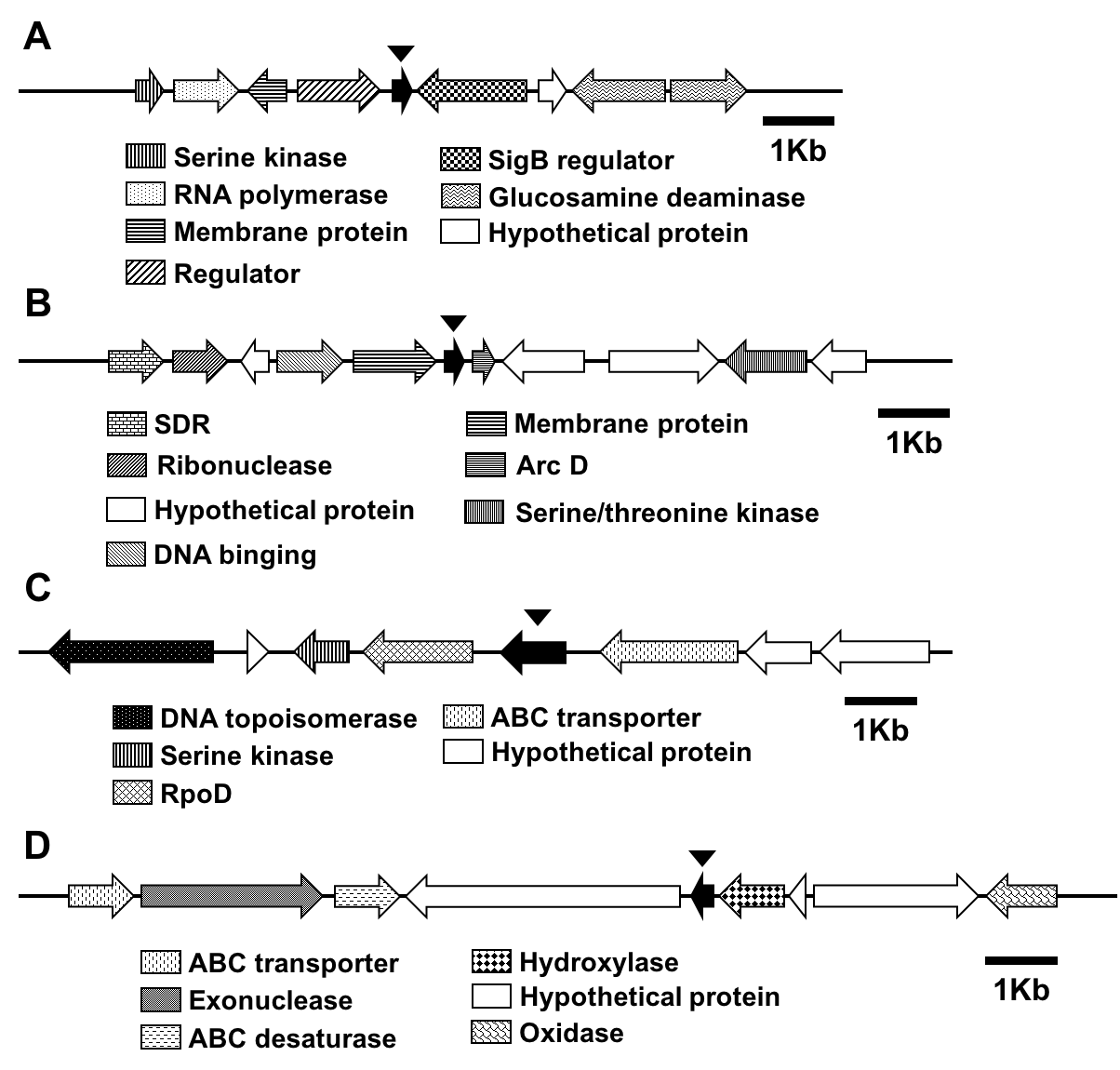


Fig S2. Plant protection assay with bacterial mutants. Plant protection bioassay with strains S4-7, S4-7Δ*wblE*, S4-7Δ*whiH*, S4-7Δ*whmD*, and S4-7Δ*wblE*2. *F. oxysporum* was inoculated as a macrospore stock (1 × 10^7^ cfu/g of soil). The bioassay was performed from day 7 to 45. The photographs were taken 45 d after treatment. A, control; B, S4-7; C, Δ*whmD*; D, Δ*wblE2*; E, Δ*whiH*; F, Δ*wblE*; G, *F. oxysporum* F-9.


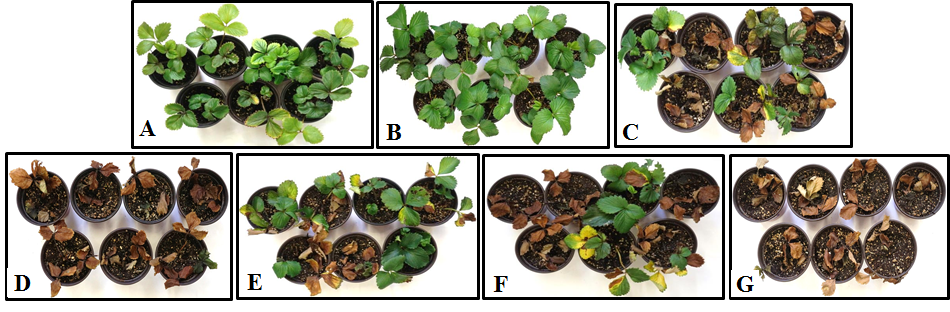


Fig S3. Number of genes identified as differentially expressed in the *wblE2* mutant grouped according to GO categories. The genes were classified with Non-supervised Orthologous Groups (eggNOG).


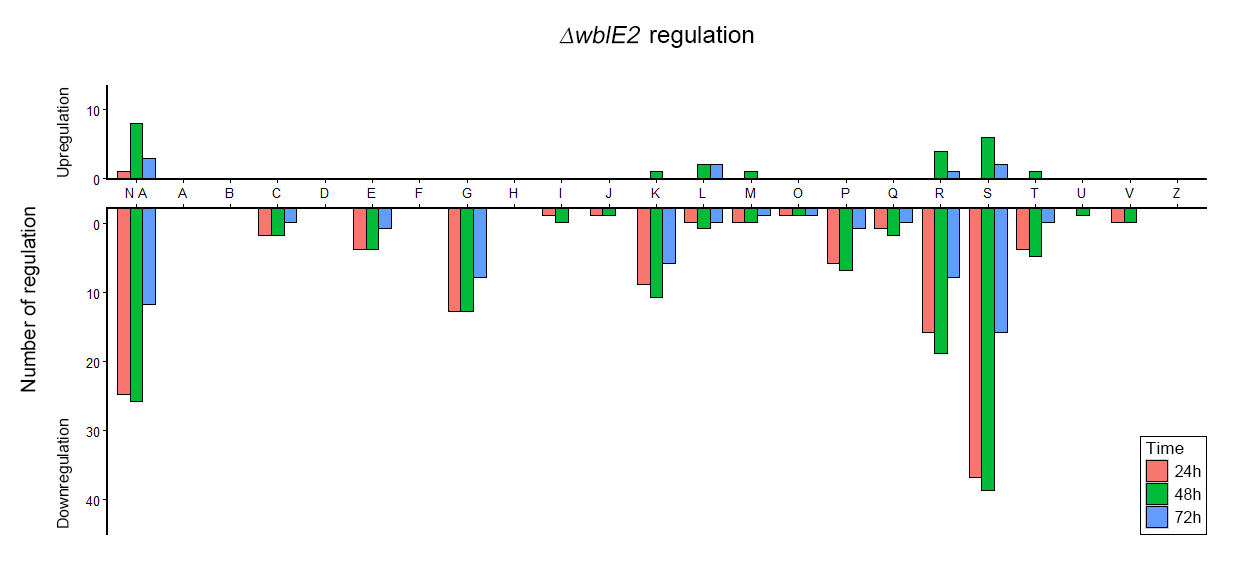


Fig S4. Validation of transcriptome results by qRT-PCR


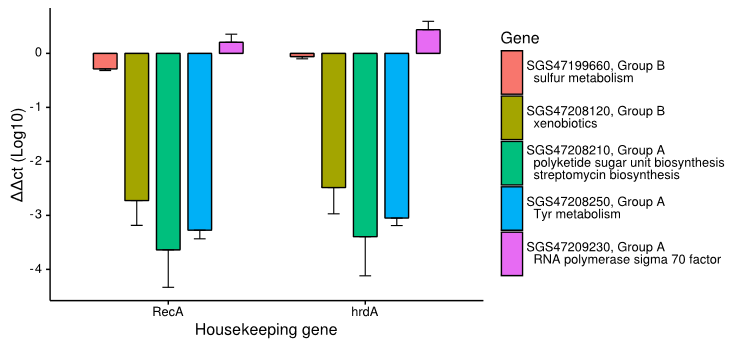

Supplement: Supplementary file 1 [file MBO3-6-na-s001.docx]
